# Anti-Melanogenesis Activity of 6-*O*-Isobutyrylbritannilactone from *Inula britannica* on B16F10 Melanocytes and In Vivo Zebrafish Models

**DOI:** 10.3390/molecules25173887

**Published:** 2020-08-26

**Authors:** Dae Kil Jang, Chau Ha Pham, Ik Soo Lee, Seung-Hyun Jung, Ji Hye Jeong, Han-Seung Shin, Hee Min Yoo

**Affiliations:** 1 Department of Food Science and Biotechnology, Dongguk University, Seoul 10326, Korea; ktcommer@hanmail.net; 2 StarlingForce Co., Ltd., Seoul 08511, Korea; 3 Microbiological Analysis Team, Group for Biometrology, Korea Research Institute of Standards and Science (KRISS), Daejeon 34113, Korea; pham.ha.chau@hotmail.com; 4 Department of Microbiology and Molecular Biology, Chungnam National University, Daejeon 34134, Korea; 5 Herbal Medicine Research Division, Korea Institute of Oriental Medicine, Daejeon 34054, Korea; knifer48@kiom.re.kr; 6 Department of Applied Marine Bioresource Science, National Marine Biodiversity Institute of Korea, Seocheon, Chungnam 33662, Korea; zebrajung@mabik.re.kr (S.-H.J.); wjdwlgpwkd27@mabik.re.kr (J.H.J.)

**Keywords:** *Inula britannica*, 6-*O*-isobutyrylbritannilactone (IBL), melanogenesis, B16F10 melanocytes, zebrafish embryos

## Abstract

A potential natural melanogenesis inhibitor was discovered in the form of a sesquiterpene isolated from the flowers of *Inula britannica*, specifically 6-*O*-isobutyrylbritannilactone (IBL). We evaluated the antimelanogenesis effects of IBL on B16F10 melanocytes and zebrafish embryos. As a result, we found that 3-isobutyl-1-methylxanthine (IBMX)-induced melanin production was reduced in a dose-dependent manner in B16F10 cells by IBL. We also analyzed B16F10 cells that were and were not treated with IBMX, investigating the melanin concentration, tyrosinase activity, mRNA levels. We also studied the protein expressions of microphthalmia-associated transcription factor (MITF), tyrosinase, and tyrosinase-related proteins (TRP1, and TRP2). Furthermore, we found that melanin synthesis and tyrosinase expression were also inhibited by IBL through the modulation of the following signaling pathways: ERK, phosphoinositide 3-kinase (PI3K)/AKT, and CREB. In addition, we studied antimelanogenic activity using zebrafish embryos and found that the embryos had significantly reduced pigmentation in the IBL-treated specimens compared to the untreated controls.

## 1. Introduction

Melanocyte is a neural crest-derived skin cell that produces the protective pigment melanin [1]. Melanin is generated by melanosomes in melanocytes, which are located in the epidermis, and is the substance that affects hair, skin, and eye color in mammals [2]. In addition, melanin biosynthesis is crucial in the body’s defense against the harmful effects to the skin caused by DNA damage and ultraviolet (UV) radiation [3,4]. However, excessive production of melanin due to frequent or excessive exposure to UV radiation can cause abnormal hyperpigmentation as a result of inflammation, age spots, freckles, lentigo senilis, and melasma [5].

Tyrosinase serves as a catalyst for the process in which l-tyrosine is hydroxylated to 3,4-dihydroxyphenylanaine (L-DOPA) in addition to the process in which this *o*-diphenol is oxidized to the corresponding quinone. On the other hand, l-dopaquinone services as a rate-limiting enzyme in the process of melanin synthesis [6]. Also present in melanosomes with tyrosinase are tyrosinase-related protein 1 (TRP1) and tyrosinase-related protein 2 (TRP2), which are important catalysts for reactions that produce eumelanin, a dark brown-black insoluble polymer [7,8]. α-melanocyte-stimulating hormone (α-MSH) and IBMX are key physiologic inducers of melanogenesis [9,10]. α-MSH and IBMX are known to stimulate tyrosinase activity through the cyclic adenosine monophosphate (cAMP) pathway [11]. Furthermore, α-MSH binds to melanocortin-1 receptors (MC1R) on cell surfaces to activate the protein kinase A (PKA) pathway, phosphorylating the CREB transcription factor and inducing the expression of microphthalmia-associated transcription factor (MITF) [12]. Several signaling pathways regulate the activity of MITF: not only cAMP but also MAPK/ERK Kinase (MEK) [13]. By binding to the M-box (AGTCATGTGCT) of tyrosinase distal elements (TDEs), MITF is able to regulate the expressions of TRP1 and TRP2 [14,15].

Tyrosinase levels are an important part of the melanin synthesis process, as decreased tyrosinase production correlates to reduced melanin pigmentation, which leads to skin whitening [16]. Popular whitening agents, such as arbutin, kojic acid, and ascorbic acid, have been used to inhibit melanogenesis by down-regulating tyrosinase activity [16]. However, various anti-melanogenic agents have serious side-effects, such as vitiligo and albinism [17,18]. Therefore, natural melanogenesis inhibitors are in high demand as researchers strive to prevent hyperpigmentary disorders, develop therapies, and contribute to cosmetic industries [19].

*Inula britannica* Linnaeus of the *Asteraceae* family, also known as “Xuan-Fu-Hua” in China, is widely used in traditional Chinese medicine for anticancer, antioxidant, antibacterial, anti-inflammation, neuroprotective, and hepatoprotective purposes [20]. A previous study reported that a flower extract of *I. britannica* markedly reduced melanin production in B16F10 melanoma cells [21]. Through a further study, we discovered a new sesquiterpene called inularin structure and investigated its antimelanogenic activity [22]. The aim of this study was to discover safe and effective natural compounds for skin whitening and preventing abnormal hyperpigmentation. Here, we discovered that 6-*O*-isobutyrylbritannilactone (IBL) exhibits antimelanogenic effects in B16F10 cells, and also that it affects melanin production in embryonic zebrafish.

## 2. Results and Discussion

### 2.1. Extraction and Isolation of IBL

**Figure 1:**
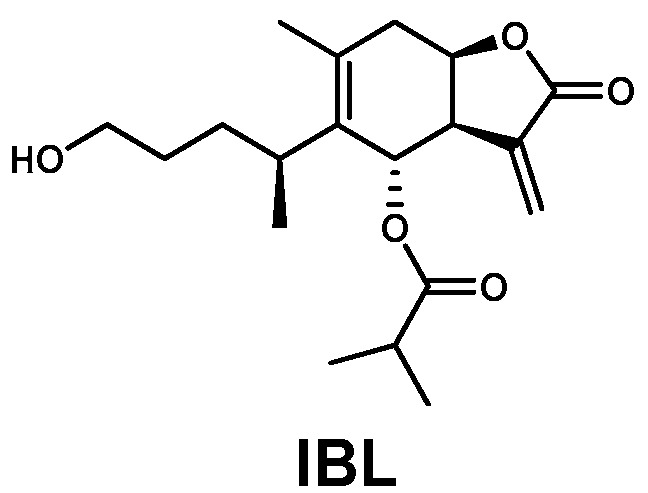
Chemical structure of 6-*O*-isobutyrylbritannilactone (IBL) isolated from the flower of *I. britannica*.

In our previous study, silica gel chromatography was performed on an ethanol extract of *I. britannica* flowers, which resulted in four fractions (A–D) based on thin-layer chromatography (TLC) data. Fraction D, which markedly reduced melanin production in B16F10 cells, was subjected to further chromatography guided by its antimelanogenic effects, leading to the isolation of an active compound [22]. This compound was an amorphous white powder with a molecular ion peak at *m*/*z* 337 [M + H]^+^ on electron ionization mass spectrometry (ESIMS), corresponding to a molecular formula of C_19_H_28_O_5_. By comparing its physicochemical and spectral data with those in the literature [23,24], this compound was identified as 6-*O*-isobutyrylbritannilactone (IBL, Figure 1).

### 2.2. Cytotoxicity of IBL in B16F10 Cells

**Figure 2:**
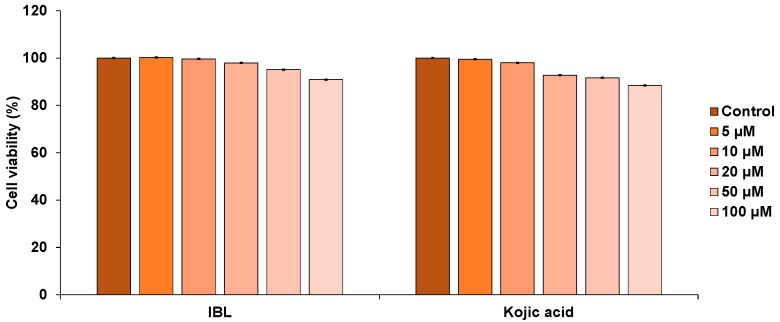
Effect of IBL on cell viability in B16F10 cells. After incubation of B16F10 melanoma cells with various concentrations of IBL (5–100 µM) in a 96-well plate for 24 h, cell viability was determined through the MTS assay. Values are represented as means ± SEM (*n* = 3).

To assess cell viability, we used the 3-(4,5-dimethylthiazol-2-yl)-2,5-diphenyltetra-zolium bromide (MTT) assay. B16F10 cells were treated with various concentrations of IBL (5–100 µM) to investigate whether IBL exhibited cytotoxic effects on melanocytes. According to the results, IBL did not significantly affect cell viability (Figure 2). Thus, we decided to investigate the melanogenesis inhibitory effect of IBL, since this IBL was found to exhibit less cytotoxicity at high concentrations, which is a beneficial property for candidate whitening agents.

### 2.3. Effects of IBL on Melanin Synthesis

**Figure 3:**
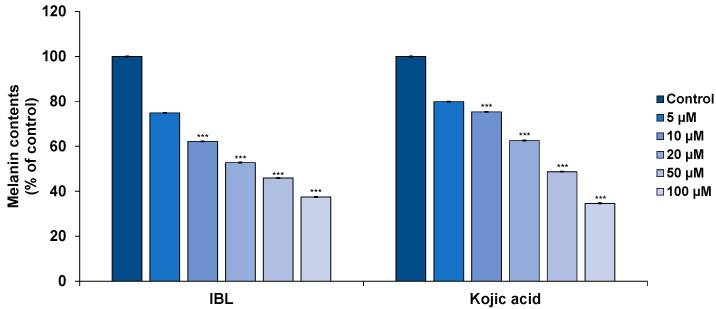
Effect of IBL on melanin content in B16F10 cells. B16F10 cells were stimulated with 100 µM IBMX for 24 h after pretreatment with the indicated concentrations of IBL (5–100 µM) or kojic acid (5–100 µM) for 48 h. The melanin content was measured at 405 nm using a microplate reader. Values are represented as means ± SEM. (*n* = 3, *** *p* ≤ 0.001).

The melanogenesis activity of IBL on melanin synthesis was assessed by treating B16F10 cells with IBL for 72 h, then treating with IBMX before measuring the melanin content. As a result of the IBL treatment, the melanin content in the IBMX-treated cells were significantly decreased in a dose-dependent manner, whereas kojic acid exhibited slightly less inhibition compared to IBL at lower concentrations below 20 µM (Figure 3). Based on the cytotoxicity data, we concluded that the nonspecific cell toxicity of IBL was not the basis for its capacity to inhibit melanogenesis.

### 2.4. Effects of IBL on Tyrosinase Activity

**Figure 4:**
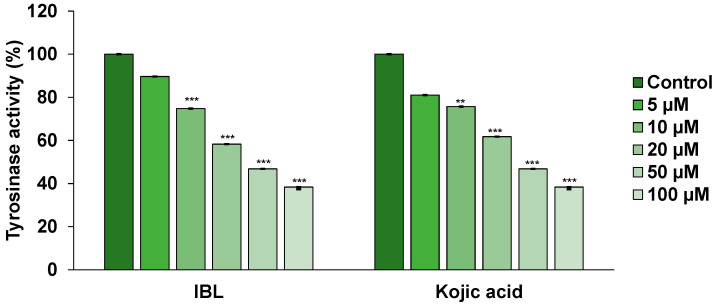
Effect of IBL on tyrosinase activity in B16F10 cells. B16F10 cells were stimulated with 100 µM IBMX for 24 h after pretreatment with the indicated concentrations of IBL (5–100 µM) or kojic acid (5–100 µM) for 48 h. Tyrosinase levels were assayed by measuring L-DOPA oxidation at 475 nm using a microplate reader. Values are represented as means ± SEM. (*n* = 3, ** *p* ≤ 0.01, *** *p* ≤ 0.001).

Tyrosinase is an enzyme that serves a key role in melanin pigment production [25]. When melanin is synthesized, it not only promotes aging of the skin, but also causes skin hyperpigmentation and dark spots. In particular, with tyrosinase being involved in the early stages of the melanin biosynthetic pathway, many whitening agents involve mechanisms of action to inhibit this enzyme [26]. Therefore, the development of whitening cosmetic materials is often focused on the development of tyrosinase inhibitors that are safe for the human body with no side effects [27]. Due to tyrosinase affecting melanin synthesis as a rate-limiting enzyme, we aimed to determine the degree to which IBL inhibits cellular tyrosinase activity in B16F10 cells. As shown in Figure 4, enzyme activity was measured through treatment with the tyrosinase enzyme inducers IBMX and IBL with concentrations of 5, 10, 20, 50, and 100 µM to investigate the inhibitory activity of IBL in cells. As a result, it was confirmed that IBL significantly decreased cellular tyrosinase activity in a dose-dependent manner in IBMX-stimulated B16F10 cells. From the above results, it can be seen that the IBL compound is a useful tyrosinase inhibitor that effectively inhibits melanin induced by IBMX. On the other hand, kojic acid was used as a control and exhibited highly similar tyrosinase inhibitory activity patterns.

### 2.5. Effects of IBL on Melanogenesis-Related Gene Expression

**Figure 5:**
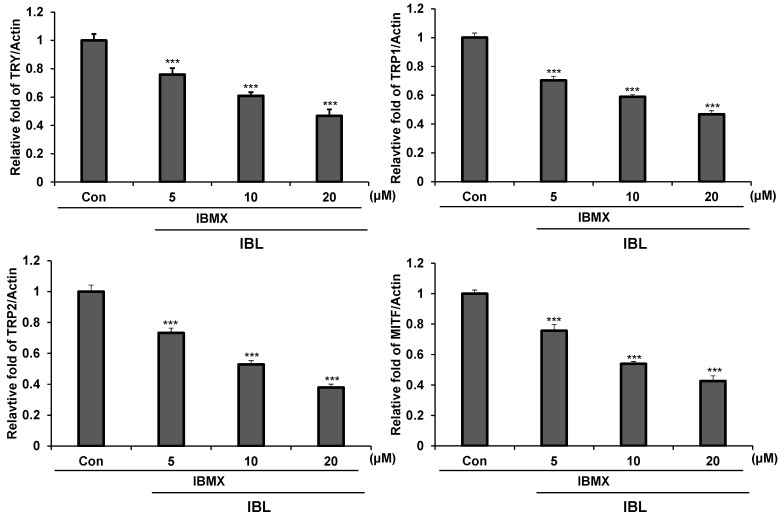
Inhibitory effects of IBL on whitening-related mRNA levels in B16F10 cells. B16F10 cells were stimulated with 100 µM IBMX for 48 h after pre-treatment with the indicated concentrations of IBL (5, 10, 20 µM) for 12 h. Real time-PCR was performed using gene specific primers for tyrosinase, Trp1, Trp2, and MITF, The expression levels were normalized to that of actin. Values are represented as means ± SEM. (*n* = 3, *** *p* ≤ 0.001).

It is important to understand whether if there is a relation between melanin synthesis inhibition by IBL and melanogenesis-related gene expression. Thus, we examined the mRNA levels of tyrosinase, TRP1, TRP2, and MITF via real time-quantitative PCR. As a result, we found that IBL treatment resulted in decreased mRNA levels of all of the aforementioned genes in a dose-dependent manner in IBMX-treated groups (Figure 5).

### 2.6. Effects of IBL on the Protein Expression of Tyrosinase, Trp1, Trp2, and MITF

**Figure 6:**
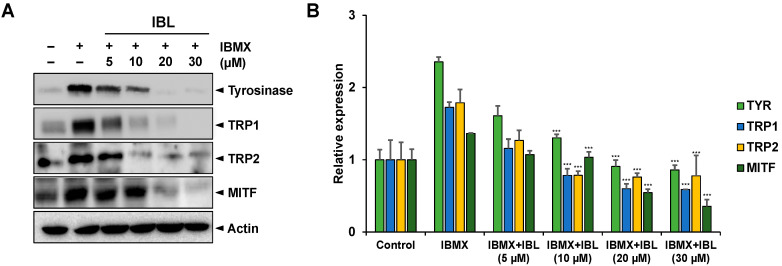
Inhibitory effects of IBL on whitening-related protein expressions in B16F10 cells. (**A**) B16F10 cells were stimulated with 100 µM IBMX for 48 h after pre-treatment with the indicated concentrations of IBL (5, 10, 20, 30 µM) for 12 h. Western blotting was performed with specific antibodies for tyrosinase, TRP1, TRP2, and MITF. (**B**) Bar graphs of the relative expressions of tyrosinase, TRP1, TRP2, and MITF. The expression levels were normalized to that of actin. Values are represented as means ± SEM. (*n* = 3, *** *p* ≤ 0.001).

To investigate whether IBL could affect melanogenic protein expression of tyrosinase, TRP1, TRP2, and MITF, we performed western blotting analysis with IBL-treated B16F10 cell lysates. In the case of TRP1 and TRP2, several studies have reported these two proteins are crucial in the melanin synthesis pathway for the following: catalyzing the process in which 5,6-dihydroxyindole-2-carboxylic acid (DHICA), which can be converted into indole-5,6-quinone carboxylic acid, is oxidized, as well as the process in which dopachrome undergoes tautomerization to form DHICA [28,29,30]. As shown in Figure 6A,B, the protein expression of tyrosinase decreased in a dose-dependent manner due to IBL treatment. Furthermore, we determined that IBL treatment resulted in the time-dependent reduction of TRP1 and TRP2 protein levels in tandem with tyrosinase and MITF.

Interestingly, it is known that MITF at the serine 73 residue is phosphorylated upon the activation of ERK signaling, which results in MITF being ubiquitinated and degraded. This is the ERK pathway’s feedback mechanism involved in the regulation of melanin production [31,32]. Based on our results, IBL treatment resulted in dose-dependent reductions in the mRNA levels of all melanogenesis-related mRNA levels. In addition, 20 µM IBL treatment resulted in completely inhibited expressions of melanogenesis-related protein levels (Figure 6). This suggests that IBL may affect melanogenic protein expression not only through transcription levels but also the post-translational modification (PTM) of melanogenesis-related proteins such as MITF and TYR [33,34,35].

### 2.7. Effects of IBL on the Expression of Melanogenesis-Related Proteins

**Figure 7:**
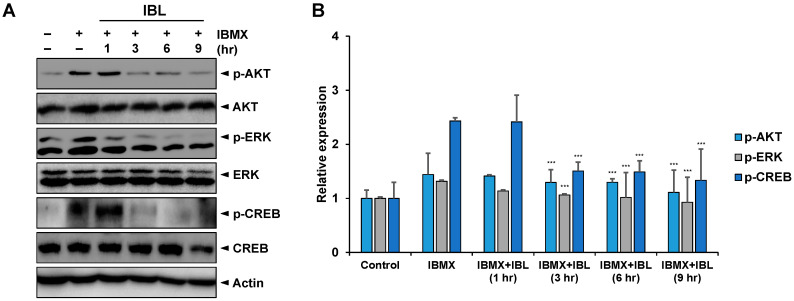
(**A**) Effects of IBL on the protein expressions of p-AKT, p-ERK, and p-CREB in B16F10 cells. B16F10 cells were pre-treated with 100 µM IBMX for 48 h and then exposed to IBL for the indicated time periods. Whole cell lysates were subjected to Western blot analysis using specific antibodies against phospho-AKT, AKT, phospho-ERK, ERK, phospho-CREB, and CREB. Equal protein loading was confirmed by actin. (**B**) Bar graphs of the relative expressions of phospho-AKT, phospho-ERK, and phospho-CREB. The expression levels were normalized to that of actin. Values are represented as means ± SEM. (*n* = 3, *** *p* ≤ 0.001).

Another key element in melanogenesis is the activation of Akt signaling [36,37]. In addition, microphthalmia-associated transcription factor (MITF) also serves to control proteins involved in melanogenesis. This transition factor is regulated by mitogen-activated protein kinase (MAPK) signaling pathways, such as the ERK pathway, which also subsequently results in increased tyrosinase expression [38,39]. ERK activation signals increase phosphorylated CREB and consequent MITF expression [40,41,42]. Thus, we investigated the ways in which IBL affected the phosphorylation of Akt and ERK. As shown in Figure 7A,B, IBMX activated Akt and ERK, and both signaling pathways exhibited time-dependent inhibition due to IBL treatment, the effect of which was sustained for at least nine hours. Moreover, the phosphorylation of CREB was also inhibited by IBL. The results demonstrate that IBL suppresses IBMX-induced melanogenesis through the inactivation of multiple signaling pathways.

The IBL compound acts as a tyrosinase inhibitor that effectively inhibits melanin induced by IBMX (Figure 4). Moreover, IBL treatment resulted in both decreased mRNA and protein levels of tyrosinase and related molecules in IBMX-treated groups (Figure 5 and Figure 6). One of the possible reasons behind the inhibition of tyrosinase activity is the decreased expression of tyrosinase. Previous studies showed that 1-*O*-acetylbritannilactone from *Inula Britannica* inhibits tyrosinase activity by suppressing tyrosinase expression via ERK and Akt signaling rather than by directly inhibiting catalytic activity [21]. Likewise, there are several studies that report melanogenesis is suppressed as tyrosinase activity is inhibited by accelerating the proteasomal degradation of tyrosinase [33,43]. On the other hand, previous studies have shown that natural product (NP) compounds are capable of interacting with multiple cellular targets and targeting multiple signaling pathways, thus highlighting the potential of NP compounds as multi-target agents [27,44,45,46]. The ERK signaling pathway phosphorylates CREB and subsequently activates MITF to promote TYR, TRP1, and TRP2 transcription. Moreover, the activation of the AKT signaling pathway leads to increased MITF activity [27]. IBL inhibits melanogenesis by regulating AKT-, ERK-, and CREB-mediated pathways and also inhibits not only tyrosinase but also MITF, TRP1, and TRP2 (Figure 5, Figure 6 and Figure 7). Therefore, our results could suggest that IBL, which is a natural product compound, could be a potential multi-target therapeutic agent for whitening and preventing abnormal hyperpigmentation.

### 2.8. Effects of IBL on Melanin Pigmentation in Zebrafish Embryos

**Figure 8:**
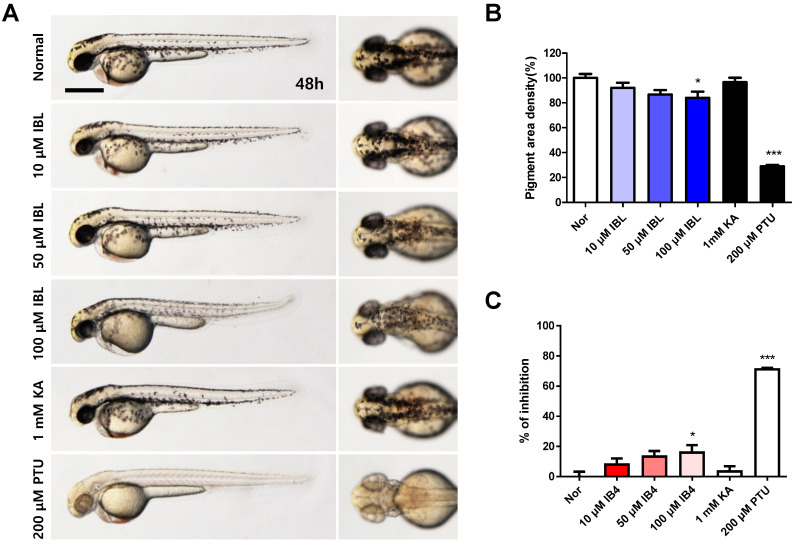
Effect of IBL on melanin production in developing zebrafish embryos. Zebrafish embryos were treated with IBL (10, 50, or 100 µM), kojic acid (KA, 1 mM), phenylthiourea (PTU, 200 µM), or 0.1% (v/v) DMSO (Normal). (**A**) Pigmentation in the embryos was observed under a stereomicroscope (lateral and dorsal views) at 48 hpf. Scale bar: 0.5 mm. (**B**,**C**) The pigmented area density and inhibition rate was normalized to that of the control embryos using the Image J software (*n* = 8). The values are represented as means ± SEM from three independent experiments. * *p* < 0.05 vs. Control, *** *p* < 0.001 vs. Control. 0.1% A DMSO-treated embryo was set as the normal.

Based on a previous method [27], we conducted a zebrafish in vivo assay for anti-pigmentation. This involved tests that were conducted on wild-type zebrafish embryos to demonstrate the ways in which single IBL compounds affected melanogenesis in an animal model. Zebrafish embryos at 10 h post-fertilization (hpf) were subjected to IBL treatment of various concentrations and were observed at 48 hpf (Figure 8A). The positive control involved the use of 1 mM kojic acid for anti-melanogenic effects in addition to 200 µM of the well-known tyrosinase inhibitor Phenylthiourea (PTU). Compared to DMSO treatment, PTU treatment resulted in clearer melanogenesis inhibition in developing zebrafish embryos (Figure 8A,B). Compared to the untreated control group, IBL at 10, 50, and 100 µM reduced pigmentation by approximately 8%, 13%, and 16%, respectively (Figure 8C). PTU reduced pigmentation by 71% at 200 µM, whereas kojic acid did not produce any significant effects at 200 µM (data not shown), although it did reduce pigmentation by 3% at 1 mM (Figure 8C).

In the case of kojic acid that was used as a positive control, high activity was not observed in previously reported papers either, and PTU acted as a strong pigmentation inhibitor in zebrafish development [47,48]. This is because kojic acid is a topically applied depigmenting agent that exerts its effect by acting as a slow-binding, competitive inhibitor of tyrosinase. As a result of the zebrafish experiment, IBL was observed to exhibit higher melanogenesis inhibition activity compared to the kojic acid positive control. Thus, IBL could be a potential natural agent for anti-melanogenesis.

## 3. Materials and Methods

### 3.1. Plant Material

Flowers of *I. britannica* were purchased from a traditional herbal medicine store in Daejeon, Republic of Korea, in April 2018 and identified by Prof. Ki Hwan Bae (College of Pharmacy, Chungnam National University, Republic of Korea). A voucher specimen (IB2018-010) has been deposited in the herbarium of the Korea Institute of Oriental Medicine, Republic of Korea.

### 3.2. Extraction and Isolation

Air-dried flowers of *I. britannica* (200 g) were extracted in ethanol (2 L) at 80 °C for 3 h, filtered, and concentrated to yield an ethanol extract (12 g). The extract (10 g) was subjected to silica gel column chromatography (50 × 10 cm) using a methylene chloride-methanol (1:0→0:1) gradient solvent system. The column chromatographic fractions were combined to give three final fractions (A, 1.8 g; B, 3.2 g; C, 2.2 g) based on TLC data. Sesquiterpene-rich fraction B was subjected to RP-18 column chromatography (50 × 4 cm) using a methanol-water (20:80→90:10) gradient solvent system; three subfractions (B1–B3) were obtained. Fraction B2 (0.6 g) was further chromatographed on an RP-18 column (50 × 3 cm) eluted with a methanol-water (40:60→80:20) gradient to obtain 6-*O*-isobutyrylbritannilactone (80 mg).

### 3.3. 6-O-Isobutyrylbritannilactone

White amorphous powder; [α]_D_^25^ + 90° (*c* 0.1, MeOH); UV (MeOH) λ_max_ 205 nm; IR (KBr) *ν*_max_ 3495, 2926, 1728, 1722, 1640, 1412, 1352, 1260, 1029 cm^−1^; ESIMS *m*/*z* 337 [M + H]^+^; ^1^H NMR (400 MHz, CD_3_OD) δ 6.30 (1H, d, *J* = 2.4 Hz, H-13a), 5.97 (1H, d, *J* = 2.4 Hz, H-13b), 5.22 (1H, d, *J* = 1.8 Hz, H-6), 5.02 (1H, m, H-8), 3.52 (1H, m, H-1a), 3.36 (1H, m, H-1b), 1.81 (3H, s, CH_3_-14), 1.14 (3H, d, *J* = 6.8 Hz, CH_3_-4′), 1.12 (3H, d, *J* = 6.8 Hz, CH_3_-3′), 0.87 (3H, d, *J* = 6.8 Hz, CH_3_-15); ^13^C NMR (100 MHz, CD_3_OD) δ 178.4 (C-1′), 172.1 (C-12), 138.5 (C-11), 135.0 (C-10), 133.8 (C-5), 125.7 (C-13), 77.4 (C-8), 70.6 (C-6), 63.0 (C-1), 44.4 (C-7), 35.7 (C-9), 35.5 (C-2′), 34.6 (C-4), 32.4 (C-2), 31.8 (C-3), 20.7 (CH_3_-14), 19.4 (CH_3_-3′), 19.4 (CH_3_-4′), 19.2 (CH_3_-15).

### 3.4. Cell Cultures

B16F10 melanoma cells were obtained from the Korea Cell Line Bank (KCLB). Cells were cultured at 37 °C in a humidified atmosphere with 5% CO_2_ in Dulbecco’s Modified Eagle Medium (DMEM, phenol red-free) supplemented with 10% fetal bovine serum (FBS), 100 µg/mL streptomycin, and 100 U/mL penicillin. DMEM, FBS, and Phosphate-buffered saline (PBS) were purchased from Thermo Fisher Scientific (Waltham, MA, USA).

### 3.5. Cell Viability Assay

Cell viability was determined using the CellTiter 96 AQueous One Solution Cell Proliferation Assay Kit containing the 3-(4,5-dimethylthiazol-2-yl)-5-(3-carboxymethoxyphenyl)-2-(4-sulfophenyl)-2H-tetrazolium (MTS) assay, as per the manufacturer’s protocol. B16F10 cells were seeded at a density of 2 × 10^5^ cells/well in a 96-well plate and incubated at 37 °C for 24 h. The *I. britannica* compounds were then added to individual wells at various concentrations. After incubation, 20 µL of MTS solution was added to each well and the cells were incubated at 37 °C for 2 h. The supernatant was then removed, and 0.1 mL of dimethylsulfoxide (DMSO) was added to dissolve the crystals in each well. The signals were detected with absorbance readings at 570 nm using an EnSpire multimode plate reader (Perkin Elmer, Waltham, MA, USA). Cell viability was calculated as the percentage of viable cells relative to the control group.

### 3.6. Measurement of Melanin Content

The extracellular melanin content was measured as described in a previous study with some modifications [21]. B16F10 melanocytes were incubated at a density of 3 × 10^5^ cells in a 96-well plate for 24 h. 50 µM of 3-isobutyl-1-methylxanthin (IBMX) was then added and the cells were treated with or without chemicals in phenol red-free DMEM for 72 h. Following the treatment, the cells were detached and centrifuged at 5000× *g* for 5 min. The cell pellets were then solubilized in 1 N NaOH at 95 °C for 15 min. The optical densities were measured at 420 nm using an EnSpire multimode plate reader. The melanin content values were expressed as percentages of the untreated control value.

### 3.7. Tyrosinase Activity

Intracellular tyrosinase activity was measured as described in a previous study [21], with some modifications. Once the cells were incubated with I. britannica compounds at various concentrations (1–30 µM) for 72 h, the cells were washed with PBS then lysed with PBS (pH 6.8) containing 1% Triton X-100. The cells were then disrupted via vortexing, and the lysates were clarified via centrifugation at 10,000× *g* for 5 min. After protein quantification and the adjustment of the protein concentrations, 90 µL of each lysate was placed in each well of a 96-well plate, and 10 µL of 10 mM L-DOPA was added to each well. During incubation at 37 °C, the absorbance was measured every 10 min at 475 nm using an EnSpire multimode plate reader.

### 3.8. Western Blot Analysis

Once the cells were harvested, the cell pellets were lysed in radioimmunoprecipitation (RIPA) buffer containing 1% NP-40, 1% sodium deoxycholate, and protease inhibitor (PI) cocktail on ice for 30 min. This was followed by centrifugation at 13,000 rpm for 30 min at 4 °C. The resulting supernatants were subsequently collected. Proteins were separated using 8% to 15% SDS polyacrylamide gel electrophoresis (SDS-PAGE) gels and transferred onto a polyvinylidene difluoride (PVDF) membrane. The membranes were blocked with 5% skim milk for 1 h at room temperature, followed by incubation with a primary antibody. Anti-Tyrosinase, anti-TRP1, anti-TRP2, anti-MITF, anti-phospho AKT, anti-AKT, anti-phospho ERK, anti-ERK, anti-phospho CREB, anti-CREB, and anti-actin antibodies were purchased from Santa Cruz Biotechnology (Santa Cruz, CA, USA). Membranes were washed with Tris-buffered saline containing 0.1% Tween-20 (TBST) and incubated with donkey anti-rabbit or anti-mouse horseradish peroxidase (HRP)-conjugated IgG secondary antibody for 2 h. Protein bands were detected by an ImageQuant LAS 4000 mini (Fujifilm, Tokyo, Japan) and visualized using an image analysis program (Multi Gauge Ver. 3.0, Fujifilm, Tokyo, Japan).

### 3.9. Reverse Transcription-Polymerase Chain Reaction

Total RNA was isolated from 48-h IBL-treated cells using the RNeasy Mini Kit (Qiagen, Hilden, Germany). Each PCR reaction was performed using the Maxima SYBR Green/ROX qPCR master mix (Thermo Fisher Scientific, Waltham, MA, USA). Quantitative RT-PCR analysis was also performed on a StepOnePlus Real-Time PCR system (Thermo Fisher Scientific, Waltham, MA, USA).

### 3.10. Maintenance of Zebrafish

Adult zebrafish were purchased from a commercial aquarium store and 15~20 fish were reared in a water circulation tank. Fish were maintained at 28.5 °C temperature and in a 14/10 h light/dark cycle. Zebrafish embryos were obtained via natural mating and developed in egg water, which consisted of 60 µg/mL Sea Salt (Sigma-Aldrich, St. Louis, MO, USA) in distilled water.

### 3.11. Chemical Treatment of Zebrafish Embryos and Imaging

Phenylthiourea (PTU, Sigma-Aldrich, St. Louis, MO, USA) was dissolved in the egg water. 1 mM Kojic acid and 200 µM PTU were used for the positive control in all experiments. IBL was dissolved in DMSO (5,5-dimethyl-1-pyroline-*N*-oxide). For the anti-pigmentation effect test, 10 hpf (hour post fertilization) zebrafish embryos were arrayed in a 24-well plate (eight individuals per well) containing 2 mL egg water. Normal (DMSO), PTU, kojic acid, and IBL were added to wells containing the zebrafish embryos in a 28.5 °C incubator. For imaging, the embryos were anesthetized with tricaine (MS-220, Sigma-Aldrich, St. Louis, MO, USA) and mounted on 3% methyl cellulose (Kanto Chemical Co., Tokyo, Japan). The mounted embryos were imaged with a SMZ25 stereomicroscope and a digital camera system (Nikon Instruments, Tokyo, Japan).

### 3.12. Quantitative Measurement of Melanocytes and Statistical Analysis

The proportion of melanocytes was determined using the Image J software (NIH), using equal-sized boxes of grayscale images for the dorsal view of the anterior part. The quantitative value was calculated as the percentage of black proportions per box image. To assess the significance of the differences between the normal and experimental groups, all statistical data was obtained through one-way ANOVA with Dunnett’s Multiple Comparison Test using Graphpad Prism. The significance level was set as * *p* < 0.05 versus the normal (DMSO) group and the data were represented as means ± SEM (standard error of mean).

### 3.13. Statistical Analysis

Statistical analysis was performed using GraphPad Prism 5 (GraphPad Software Inc., La Jolla, CA, USA), and the data are presented as means ± SEM. The results were further analyzed through the student’s *t*-test, and *p* values less than 0.05 were considered statistically significant.

## 4. Conclusions

In this study, a sesquiterpene, specifically 6-*O*-isobutyrylbritannilactone (IBL), was isolated from the flowers of *Inula britannica*. According to the investigation results regarding the whitening activity of IBL, it was confirmed to be a highly safe compound as no cytotoxicity was observed at high concentrations. Concentration-dependent intracellular tyrosinase inhibitory activity was observed, and the results indicated high inhibitory activity and melanin synthesis inhibition when treated with IBL. The mRNA expression levels and proteins related to melanin synthesis were analyzed by RT-PCR and western blotting to determine the whitening mechanism of IBL. According to the results, the expressions of tyrosinase, tyrosinase-related protein 1 (TRP1), and tyrosinase-related protein 2 (TRP2), which are the key rate-limiting enzymes of the melanogenesis process, and the expression of the transcription factor MITF were inhibited both mRNA and proteins, which results in whitening activity. IBL inhibits melanogenesis by regulating AKT-, ERK- (MAPK), and CREB-mediated pathways. This whitening effect was also observed through an in vivo zebrafish model, with a significant reduction in melanin pigments resulting from IBL treatment. Thus, IBL could be an effective antimelanogenic agent for skin whitening.
